# Supplementary figures and images for: DegP Initiates Regulated Processing of Filamentous Hemagglutinin in Bordetella bronchiseptica
Source: mBio. 2021 Jun 29;12(3):e01465-21. doi: 10.1128/mBio.01465-21 (PMC8263021; doi:10.1128/mBio.01465-21)

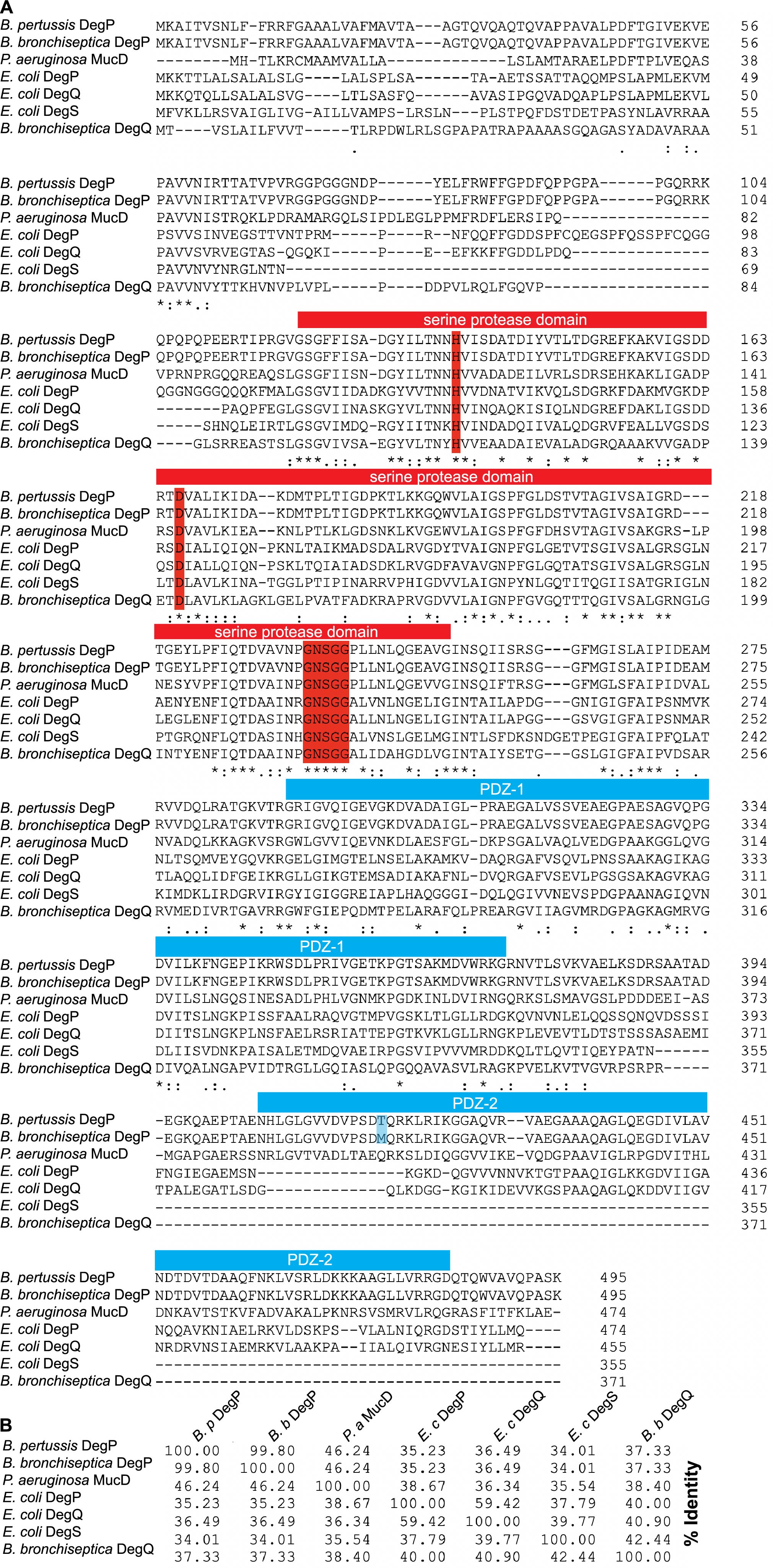

Supplement: FIG S1 [file mbio.01465-21-sf001.jpg]
