# Supplementary material for: DegP Initiates Regulated Processing of Filamentous Hemagglutinin in Bordetella bronchiseptica
Source: mBio. 2021 Jun 29;12(3):e01465-21. doi: 10.1128/mBio.01465-21 (PMC8263021; doi:10.1128/mBio.01465-21)
Supplement: FIG S2 [file mbio.01465-21-sf002.pdf]

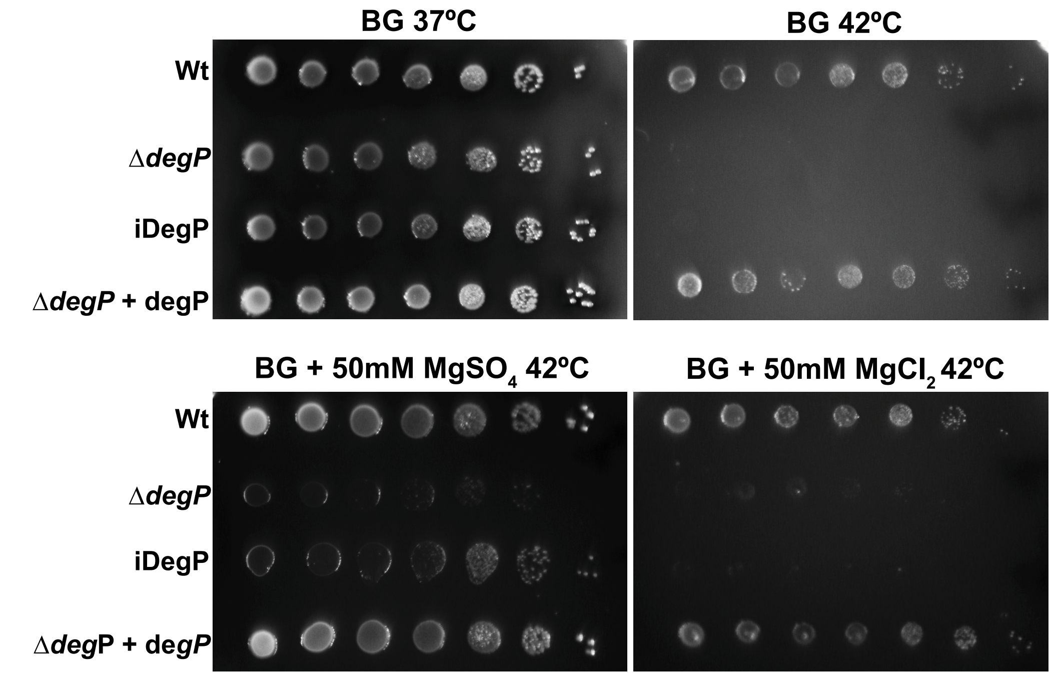

BG 37ºC
BG 42ºC
BG + 50mM MgSO4 42ºC
BG + 50mM MgCl2 42ºC
Wt
ΔdegP
iDegP
ΔdegP + degP
Wt
ΔdegP
iDegP
ΔdegP + degP
