# Supplementary material for: DegP Initiates Regulated Processing of Filamentous Hemagglutinin in Bordetella bronchiseptica
Source: mBio. 2021 Jun 29;12(3):e01465-21. doi: 10.1128/mBio.01465-21 (PMC8263021; doi:10.1128/mBio.01465-21)
Supplement: FIG S3 [file mbio.01465-21-sf003.pdf]

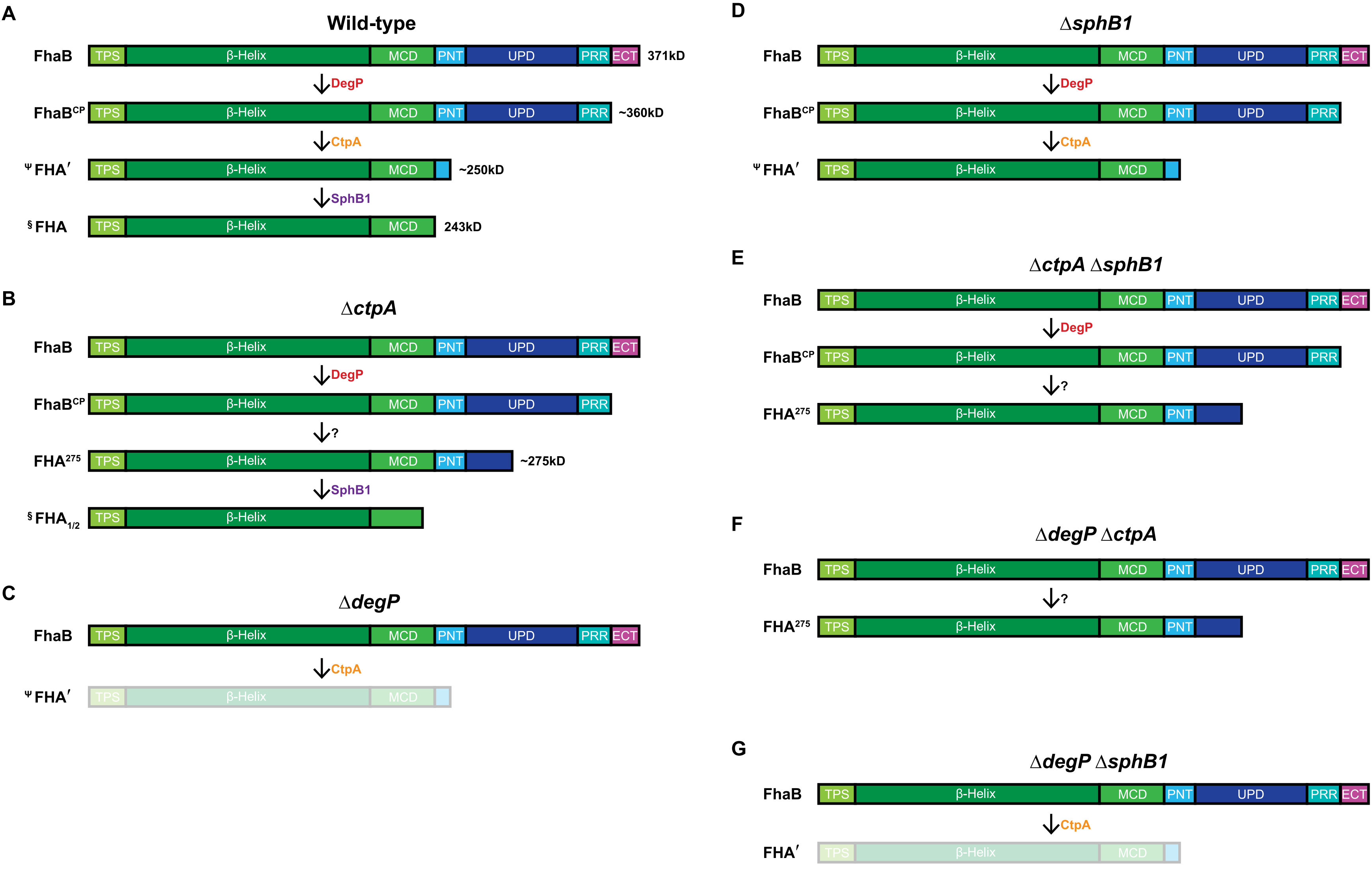

A
Wild-type
FhaB
TPS
β-Helix
MCD
PNT
UPD
PRR
ECT
371kD
DegP
FhaBCP
~360kD
CtpA
ΨFHA′
~250kD
SphB1
§FHA
243kD
B
ΔctpA
?
FHA275
~275kD
§FHA1/2
C
ΔdegP
D
ΔsphB1
E
ΔctpA ΔsphB1
F
ΔdegP ΔctpA
G
ΔdegP ΔsphB1
FHA′
