# Supplementary material for: DegP Initiates Regulated Processing of Filamentous Hemagglutinin in Bordetella bronchiseptica
Source: mBio. 2021 Jun 29;12(3):e01465-21. doi: 10.1128/mBio.01465-21 (PMC8263021; doi:10.1128/mBio.01465-21)
Supplement: TABLE S1 [file mbio.01465-21-st001.docx]

| **Bacterial strains** | **Description** | **Reference** |
| --- | --- | --- |
| RB50 | *Bordetella bronchiseptica* Complex I strain, isolated from naturally infected rabbit respiratory tract | 1 |
| RBX11 | RB50 containing a deletion of *fhaS* codons 4-3203 | 2 |
| RBX11 Δ*ctpA* | RBX11 containing an in-frame deletion of *ctpA* codons 7-477 created using a pSS4245-based plasmid | 3 |
| RBX11 Δ*sphB1* | RBX11 containing a deletion of codons 5-1035 of *sphB1* | 4 |
| RBX11 Δ*degP* | RBX11 containing an in-frame deletion of *degP* codons 6-491using a pSS4245-based plasmid | This study. |
| RBX11 Δ*degP* + *degP* | RBX11 Δ*degP* containing an integrated plasmid at the Tn7 site driving expression of *degP* via the constitutive S12 promoter | This study. |
| RBX11 Δ*sphB1* Δ*degP* | RBX11 HA-PRR containing a deletion of *sphB1* codons 5-1035 with an an in-frame deletion of *degP* codons 6-491using a pSS4245-based plasmid | This study. |
| RBX11 Δ*ctpA* Δ*degP* | RBX11 containing an in-frame deletion of *ctpA* codons 7-477 created using a pSS4245-based plasmid with an an in-frame deletion of *degP* codons 6-491using a pSS4245-based plasmid | This study. |
| RBX11 iDegP | RBX11 carrying a point mutation on the chromosome in *degP* such that the catalytic serine at position 237 is changed to an alanine | This study. |
| RBX11 ∆*envC* | RBX11 carrying an in-frame deletion in BB0299 (*envC*) codons 2-1562 | This study. |
| RBX11 ΩBB1398 | RBX11 containing a pUC18 plasmid integrated 253 bp into the BB1398 ORF | This study. |
| RBX11 ΩBB1721 | RBX11 containing a pUC18 plasmid integrated 401 bp into the BB1721 ORF | This study. |
| RBX11 ∆BB2212 | RBX11 containing an in-frame deletion of codons 6-274 of BB2112 | This study. |
| RBX11 ΩBB2462 | RBX11 containing a pUC18 plasmid integrated 289bp into the BB2562 ORF | This study. |
| RBX11 ∆BB3068 | RBX11 containing an in-frame deletion of codons 6-141 of BB3068 | This study. |
| RBX11 ΩBB3749 (*degP*) | RBX11 containing a pUC18 plasmid integrated 276 bp into the *degP* ORF | This study. |
| RBX11 ΩBB3990 | RBX11 containing a pUC18 plasmid integrated 355 bp into the BB3990 ORF | This study. |
| RBX11 ΩBB4867 | RBX11 containing a pUC18 plasmid integrated 334bp into the BB4867 ORF | This study. |
| RBX11 ∆*rpoE* | RBX11 carrying an in-frame deletion of codons 13-191 of *rpoE* | This study. |
| RBX11 ∆*degP* ∆*rpoE* | RBX11 ∆*degP* carrying an in-frame deletion of codons 13-191 of *rpoE* | This study. |
| RBX11 iDegP ∆*rpoE* | RBX11 iDegP carrying an in-frame deletion of codons 13-191 of *rpoE* | This study. |
| RBX11 HA-PRR | RBX11 containing an insertion of nine codons that encode the HA epitope (YPYDVPDYA) following *fhaB* codon 3375*.* | 5 |
| RBX11 HA-PRR Δ*ctpA* | RBX11 HA-PRR containing an in-frame deletion of ctpA codons 7-477 created using a pSS4245-based plasmid | 4 |
| RBX11 HA-PRR Δ*degP* | RBX11 HA-PRR containing an in-frame deletion of *degP* codons 6-491using a pSS4245-based plasmid | This study. |
| RBX11 HA-PRR Δ*degP + degP* | RBX11 HA-PRR *ΔdegP* with an integrated plasmid at the Tn7 site driving expression of *degP* via the constitutive S12 promoter | This study. |
| RBX11 ΔECT | RBX11 HA-PRR containing an in-frame deletion of *fhaB* codons 3613-3710 | 5 |
| RBX11 HA-PRR ΔECT Δ*ctpA* | RBX11 HA-PRR ΔECT containing an in-frame deletion of *ctpA* codons 7-477 created using a pSS4245-based plasmid | This study. |
| RBX11 ΔECT Δ*degP* | RBX11 HA-PRR containing an in-frame deletion of degP codons 6-491using a pSS4245-based plasmid | 3 |
| RBX11 HA-PRR Δ*sphB1* Δ*ctpA* | RBX11 HA-PRR Δ*sphB1* containing an in-frame deletion of *ctpA* codons 7-477 created using a pSS4245-based plasmid | 3 |
| RBX11 CT-HA | RBX11 with the nine codons that encode the HA epitope (YPYDVPDYA) inserted after fhaB codon 3710 and followed by the stop codon. Created using a pEG7-based plasmid. | 3 |
| RBX11 CT-HA Δ*ctpA* | RBX11 CT-HA containing an in-frame deletion of *ctpA c*odons 7-477 created using a pSS4245-based plasmid | This study. |
| RBX11 CT-HA Δ*degP* | RBX11 CT-HA containing an in-frame deletion of *degP* codons 6-491using a pSS4245-based plasmid | This study. |
| DH5α | *Escherichia coli* cloning strain | Gibco/Thermo Fisher |
| RHO3 | *E. coli* conjugation strain and DAP auxotroph | 6 |
| pSS4245 | Allelic exchange plasmid for *B. bronchiseptica*. Used to create in-frame deletions. | 7 |
| pSS4245 Δ*ctpA* | pSS4245 used to create in-frame deletion of *ctpA* codons 7-477 | 3 |
| pSS4245 Δ*degP* | pSS4245 used to create in-frame deletion of *degP* codons 6-491 | This study. |
| pSS4245 DegPS237A | pSS4245 used to create a point mutation on the chromosome such that the catalytic serine at position 237 in *degP* encodes an alanine | This study. |
| pSS4245 ∆*envC* | pSS4245 used to create in frame deletion of *envC* codons 2-1562 | This study. |
| pSS4245 ∆BB2212 | pSS4245 used to create in-frame deleltion of BB2112 codons 6-274 | This study. |
| pSS4245 ∆BB3068 | pSS4245 used to create in-frame deletion of BB3068 codons 6-141 | This study. |
| pSS4245 ∆*rpoE* | pSS4245 used to create in-frame deletion of *rpoE* codons 13-191 of rpoE | This study. |
| pUC18-mini-Tn7-km | plasmid for *B. bronchiseptica* used to create disruption mutations | 8 |
| pUC18 ΩBB1398 | pUC18-mini-Tn7-kmused to disrupt BB1398 at position 253 | This study. |
| pUC18 ΩBB1721 | pUC18-mini-Tn7-km used to disrupt BB1721 at position 401 | This study. |
| pUC18 ΩBB2462 | pUC18-mini-Tn7-km used to disrupt BB2462 at position 289 | This study. |
| pUC18 ΩBB3749 (*degP*) | pUC18-mini-Tn7-km used to disrupt BB37249 at position 276 | This study. |
| pUC18 ΩBB3990 | pUC18-mini-Tn7-km used to disrupt BB3990 at position 355 | This study. |
| pUC18 ΩBB4867 | pUC18-mini-Tn7-km used to disrupt BB4867 at position 334 | This study. |
| pUCS12 | pUC18-mini-Tn7-km used to integrate genes of interest driven by the S12 promoter to the *att*Tn7 site | 9 |
| pUCS12-*degP* | pUCS12 vector containing the *degP* ORF driven by the constitutively active S12 promoter that integrates at the *att*Tn7 site | This study. |
